# The effect of smoking on the duration of life with and without disability, Belgium 1997–2011

**DOI:** 10.1186/1471-2458-14-723

**Published:** 2014-07-15

**Authors:** Herman Van Oyen, Nicolas Berger, Wilma Nusselder, Rana Charafeddine, Carol Jagger, Emmanuelle Cambois, Jean-Marie Robine, Stefaan Demarest

**Affiliations:** 1 Unit of Survey, Life Styles and Chronic Diseases, Directorate Public Health and Surveillance, Scientific Institute of Public Health, Brussels, Belgium; 2 Department of Public Health, Erasmus MC, University Medical Center, Rotterdam, the Netherlands; 3 Institute for Ageing and Health, Newcastle University, Newcastle upon Tyne, United Kingdom; 4 French Institute for Demographic Studies, INED, Paris, France; 5 French Institute of Health and Medical Research, INSERM, Paris and Montpellier, France

**Keywords:** Disability free life expectancy, Disability life expectancy, Life expectancy, Health expectancy, Disability, Mortality, Smoking, Decomposition, Belgium

## Abstract

**Background:**

Smoking is the single most important health threat yet there is no consistency as to whether non-smokers experience a compression of years lived with disability compared to (ex-)smokers. The objectives of the manuscript are (1) to assess the effect of smoking on the average years lived without disability (Disability Free Life Expectancy (DFLE)) and with disability (Disability Life Expectancy (DLE)) and (2) to estimate the extent to which these effects are due to better survival or reduced disability in never smokers.

**Methods:**

Data on disability and mortality were provided by the Belgian Health Interview Survey 1997 and 2001 and a 10 years mortality follow-up of the survey participants. Disability was defined as difficulties in activities of daily living (ADL), in mobility, in continence or in sensory (vision, hearing) functions. Poisson and multinomial logistic regression models were fitted to estimate the probabilities of death and the prevalence of disability by age, gender and smoking status adjusted for socioeconomic position. The Sullivan method was used to estimate DFLE and DLE at age 30. The contribution of mortality and of disability to smoking related differences in DFLE and DLE was assessed using decomposition methods.

**Results:**

Compared to never smokers, ex-smokers have a shorter life expectancy (LE) and DFLE but the number of years lived with disability is somewhat larger. For both sexes, the higher disability prevalence is the main contributing factor to the difference in DFLE and DLE. Smokers have a shorter LE, DFLE and DLE compared to never smokers. Both higher mortality and higher disability prevalence contribute to the difference in DFLE, but mortality is more important among males. Although both male and female smokers experience higher disability prevalence, their higher mortality outweighs their disability disadvantage resulting in a shorter DLE.

**Conclusion:**

Smoking kills and shortens both life without and life with disability. Smoking related disability can however not be ignored, given its contribution to the excess years with disability especially in younger age groups.

## Background

Smoking is without doubt the single most important global cause of premature mortality. The current death toll from direct and second hand tobacco smoking in adults 30 years and over is estimated to be globally well over 5.5 million each year [1]. While at present the highest proportion of deaths attributable to tobacco are in America and Europe, the largest proportions of tobacco-related deaths in the coming decades is expected to occur in medium and low income countries [2]. Smokers may lose up to one decade of life expectancy [3,4]. However, prolonged cessation, when started early enough, reduces the risk of mortality associated with smoking by 90% or more [3-5] and hence greater mortality benefits are observed among early quitters [6]. Implementation of evidence-based tobacco control measures, such as smoke-free air laws or taxation, contribute to the avoidance of substantial numbers of premature deaths [7]. Smoking has also been associated with the incidence of chronic diseases, especially several cancers, cardiovascular diseases, and lung disease [8-10], and with the incidence of disability and poor health-related quality of life [11,12].

Although non-smoking is related to a longer life and a longer healthier life, there is no agreement in the literature on whether smoking cessation also leads to fewer years with morbidity. Some publications suggest that smoking reduces both the duration of life free of and with diseases and disability so that in the end, never smokers live the same or even more years in ill-health [8,13-17]. Other authors report that smokers have to endure in their shorter life more years and a greater proportion of their life with disability [18-20]. The first group of manuscripts suggests the need to consider a trade-off between a longer life and a longer life in ill-health [21], while the latter studies support the compression of morbidity theory that can be reached through primordial and primary prevention [22]. For public health policy, it is important to better understand this discrepancy in current literature and to better assess health gains or losses in relation to smoke reducing interventions, specifically: “Is the gap in duration of life in total and with or without disability, between never smokers and ex- or current smokers, due to differences in mortality and/or due to differences in disability?”.

The objectives of the current manuscript are therefore (1) to determine the effect of smoking on the duration of life with and without disability and (2) to estimate the contribution of the higher mortality and higher disability associated with smoking to the difference in the years lived with and without disability between smoking groups.

## Methods

### Data

To calculate Disability Free Life Expectancy (DFLE) and Disability Life Expectancy (DLE) by smoking status two sources of data are required. First, information is needed about the mortality by smoking status. This information was extracted from the mortality follow-up of the Belgian Health Interview Surveys 1997 and 2001 (HIS 1997; HIS 2001) participants. The surveys were carried out by Statistics Belgium and exempted by law from requiring ethics approval. The process of obtaining mortality follow-up information is regulated by the Belgian Commission for the Protection of Privacy. After the approval of the Commission, Statistics Belgium provided follow-up data for the HIS 1997 and HIS 2001 participants until date of death, date of emigration or until respectively 31/12/2007 and 31/12/2010. Follow-up was obtained by individual record linkage between the HIS and the National Register, a public register with details of all registered residents in Belgium, using the National Identification Number. Statistics Belgium provided the list, including the date of death, of the HIS 1997 and HIS 2001 participants who had died by the end of the follow-up period. Second, information is needed about the prevalence of disability by smoking status. This information was extracted from both surveys. The participants in these national cross-sectional surveys were selected from the National Register through a multistage stratified sample of the Belgian population aged 15 years and older. Potential participants were informed by an invitation letter with leaflet and by the interviewer that the participation to the survey is voluntary and that after given an oral consent they can stop the interview anytime or can skip a question if they felt they should not answer a particular question. The participation rate in both surveys was around 60%. The detailed methodology of the surveys is described elsewhere [23]. Data on disability and socioeconomic position were collected via face-to-face interviews, while data on smoking were provided by the participant through a self-administered questionnaire.

### Measures

#### ***Disability***

The Belgian Health Interview Surveys used the instruments proposed by the WHO-Europe working group to identify people with disability [24]. Activity restriction is used to define disability based on four dimensions: difficulties in doing any one of six Activities of Daily Living (ADL) - transfer in and out of bed, transfer in and out of chair, dressing, washing of hands and face, feeding, going to the toilet; or difficulties in mobility; continence problems; or limitations in sensory (vision, hearing) functions. Based on the severity of these different dimensions, a variable was constructed with 3 categories: severe disability, mild disability and no disability (Table 1). For people younger than 60 years, the functional domain scale of the SF-36 instrument [25] was used as a filter: (1) a score of 100 on the scale categorises the respondent as being not disabled; (2) when the score was less than 100, the disability questions were asked to the respondent, who was then classified as described in Table 1. In the manuscript we consider disability of all severity levels (mild and severe) as well as severe disability only.

**Table 1:** Definition of disability by severity

		**Mild disability**	**Severe disability**
Activity of Daily Living (ADL)	Transfer in and out bed	Ability to do the task on his/her own with difficulties	Only able to do the task with personal assistance
	Transfer in and out chair		
	Dressing		
	Washing of hands and face		
	Feeding		
	Going to the toilet		
Mobility		Ability to walk less than 200 metres without stopping	Ability to walk only a few steps or less without stopping
Continence		Loss of bladder control less than once a month	Loss of bladder control at least once a month
Sensorial functions	Vision	Inability, even with glasses, to recognise a friend at a distance of 4 metres	Inability, even with glasses, to recognise a friend at a distance of 1 metre
	Hearing	Inability, even with a hearing aid, to follow a TV program at a volume others find acceptable	Inability, even with a hearing aid, to follow a TV program at a volume others find unacceptable

### Smoking

A four-category variable was used: never smokers, ex-smokers, light smokers (less than 20 cigarette per day) and heavy smokers (20 cigarettes or more per day).

### Socio-economic position

Educational attainment was coded according to the International Standard Classification of Education (ISCED 2011) and was based on the highest level of education reached by the households’ reference person or his/her partner: lower education (ISCED 0–1), lower secondary education (ISCED 2), higher secondary education (ISCED 3) and higher education (ISCED 4–8) [26].

### Statistical methods

#### ***Mortality and disability***

For each subject, the person-years at risk for mortality were estimated up to the date of death or the end of the follow-up period. To account for the age changes during follow-up time, we used Lexis expansions of the original data with 1 year age-bands [27]. In this procedure, the observed individual follow-up times were split into periods that correspond to different current-age (or attained-age) groups. Therefore, each subject’s person-years of observation were split into several observations by expanding data by 1-year age bands. As disability, mortality and smoking are associated with age and education [16,28,29], we first estimated mortality rates and disability prevalence rates by smoking status adjusted for age and education. Poisson and multinomial logistic regression models were fitted to estimate the mortality rate and the prevalence of disability by age, gender and smoking status adjusted for socio-economic position. Lexis expansion and regression analysis were performed using Stata 10.0. The analysis accounted for the complex sampling design of the HIS.

### Life table analysis

The age specific mortality rates were used to estimate LE by gender and smoking category. DFLE and DLE at age 30 (last open age group: 85 years and plus) and partial DFLE and DLE in the age window 30–80 years (DFLE_30–80_ and DLE_30–80_) were calculated by gender and smoking category using the Sullivan method which integrates the age-specific disability prevalence into the life table [30,31]. To estimate the contribution of mortality and disability to the differences in DFLE and DLE between smoking groups, a decomposition method was used [32,33]. Differences in total life expectancy (LE), DFLE and DLE between never smokers and other smoking categories (ex-smokers, smokers, light and heavy smokers) were divided in two parts. The first component, the mortality effect, represents the differences in the expected years lived with and without disability due to a differential mortality experience between never smokers and the other smoking categories. The second component, the disability effect, represents the differences in the person-years lived with or without disability due to differences in the prevalence of disability by smoking status. Whereas differences in LE only reflect differences in mortality rates, differences in DFLE and DLE are a result of differences in age-specific mortality rates (mortality effect) and differences in the age-specific prevalence of disability (disability effect). Calculations were done using a R 2.14.2 program developed in the framework of the EHLEIS project [34] and a copy of the R program is available from W. Nusselder (w.nusselder@erasmusmc.nl). For the decomposition, including the variance estimation, the analysis by smoking intensity was only possible for the partial DFLE_30–80_ and DLE_30–80_ as there were few very old heavy smoking females.

## Results

Both the prevalence of disability and the mortality rate are higher in ex-smokers and in light and heavy smokers compared to never smokers (Tables 2 and 3). As expected, mortality rates increase with the intensity of smoking but the relationship between the prevalence of disability and the intensity of smoking is not as strong, especially for severe disability. In males, the age and education adjusted prevalence ratio (a-PR) for disability is 1.17 in ex-smokers, 1.27 in light and 1.34 in heavy smokers, whilst in females, the a-PR is 1.15 in ex-, 1.22 in light and 1.24 in heavy smokers. The prevalence of severe disability is lower, although not reaching statistical significance, in heavy smokers (a-PR = 0.83 in males; and 0.66 in females). The age and education adjusted mortality rate ratio for ex-, light and heavy smokers is respectively 1.50; 1.95 and 2.77 for males and 1.09; 1.41 and 2.67 for females.

**Table 2:** Weighted age and education adjusted (severe) disability prevalence (in %) and prevalence ratio by smoking status for those aged 30+, Health Interview Survey 1997 and 2001, Belgium

		**Disability**		**Severe disability**	
	**N**	**Prevalence (%)**	**Prevalence ratio**	**Prevalence (%)**	**Prevalence ratio**
Males					
Never smoker	1667	21.47	1	4.39	1
		(19.48; 23.46)*		(3.54; 5.25)	
Ex-smoker	2325	25.05	1.17	4.41	1.00
		(22.86; 27.24)	(1.03; 1.33)	(3.50; 5.32)	(0.76; 1.33)
Light smoker	1262	27.28	1.27	4.92	1.12
		(24.65; 29.91)	(1.11; 1.45)	(3.89; 5.95)	(0.84; 1.49)
Heavy smoker	842	28.87	1.34	3.63	0.83
		(25.88; 31.86)	(1.17; 1.55)	(2.72; 4.53)	(0.60; 1.13)
Females					
Never smoker	3376	26.60	1	5.84	1
		(26.17; 27.03)		(5.08; 6.59)	
Ex-smoker	1665	30.47	1.15	5.76	0.99
		(27.80; 33.15)	(1.03; 1.28)	(4.56; 6.96)	(0.77; 1.26)
Light smoker	918	32.58	1.22	6.40	1.10
		(27.28; 37.89)	(1.03; 1.46)	(3.66; 9.14)	(0.70; 1.71)
Heavy smoker	548	32.86	1.24	3.88	0.66
		(23.35; 42.37)	(0.92; 1.66)	(0.30; 7.45)	(0.26; 1.68)

*: 95% Confidence Interval.

**Table 3:** Weighted age and education adjusted mortality rate per 100 000 person years and mortality rate ratio by smoking status for those aged 30+, Health Interview Survey 1997 and 2001 and follow-up until respectively 31/12/2007 and 31/12/2010, Belgium

	**Observed deaths**	**Observed person years**	**Mortality rate**	**Mortality rate ratio**
Males				
Never smoker	188	19618.47	1337.87	1
			(1112.97; 1562.77)*	
Ex-smoker	541	23129.05	1736.69	1.50
			(1562.34; 1911.03)	(1.22; 1.84)
Light smoker	277	13791.72	2509.18	1.95
			(2073.85; 2944.51)	(1.52; 1.84)
Heavy smoker	120	8784.79	3999.89	2.77
			(2892.02; 5107.76)	(2.00; 3.84)
Females				
Never smoker	532	36882.12	871.25	1
			(786.18; 956.32)	
Ex-smoker	212	17891.89	1076.93	1.09
			(917.06; 1236.80)	(0.91; 1.30)
Light smoker	73	11000.82	1580.24	1.41
			(1091.34; 2069.14)	(0.98; 2.02)
Heavy smoker	54	6062.29	2453.82	2.67
			(886.06; 4021.57)	(1.34; 5.33)

*: 95% Confidence Interval.

At age 30 and compared to never smokers, ex-smokers have a shorter LE and a somewhat shorter DFLE but their DLE is about one third of a year longer (Table 4). Smokers have a shorter LE, DFLE and DLE compared to never smokers. DFLE as a proportion of LE is 74.8% in male never smokers compared to 72.7% in ex-smokers and smokers, and 65.8% in female never smokers compared to 63.6% in ex-smokers and 64.0% in smokers. Both ex-smokers and smokers are estimated to live fewer years with severe disability (DLE_S). Table 5 presents the difference in DFLE, DLE, DLE_S and LE at age 30 between ex-smokers, smokers and never smokers. A negative value indicates less years lived compared to never smokers. Each estimated difference is divided into a part due to differential age-specific mortality (mortality effect) and a part that results from a differential age-specific prevalence of disability (disability effect). Thus, compared to male never smokers, LE for male smokers is 7.87 years shorter, this difference in LE being attributable only to the mortality disadvantage that male smokers have over never smokers. Male smokers have a shorter DFLE by 6.80 years, this difference being a result of differences in both the age-specific mortality rate and age-specific disability prevalence. The mortality effect accounts for 3.67 years or 54% of the difference, while the remaining 3.13 years are due to the higher disability prevalence among smokers. Due to their disability disadvantage, smokers are expected to live 3.13 more years with disability but because of the higher mortality the disability effect is cancelled out resulting in 1.07 year shorter DLE compared to never smokers (-1.07 years = -4.21 years (mortality effect) + 3.13 years (disability effect)). In both males and females the impact of the higher mortality among smokers on the DLE outweighs the disability effect so that they live fewer years with disability. This is not the case for DLE of ex-smokers where the disability effect is larger than the mortality effect resulting in about one third of a year longer DLE. Due to a larger mortality effect, both male and female ex-smokers and smokers live shorter DLE_S, although the difference is only significant for male smokers.

**Table 4:** **Disability Free Life Expectancy (DFLE**_**30**_**), (Severe) Disability Life Expectancy (DLE(_S)**_**30**_**), Life Expectancy (LE**_**30**_**) and the % of remaining life without disability (% DFLE/LE**_**30**_**) at age 30 by smoking status, Health Interview Survey 1997 and 2001 and follow-up until respectively 31/12/2007 and 31/12/2010, Belgium**

**Smoking status**	**DFLE**_**30**_	**DLE**_**30**_	**DLE_S**_**30**_	**LE**_**30**_	**%DFLE/LE**_**30**_
Males					
Never smoker	38.30	12.89	3.00	51.19	74.82
	(36.86; 39.87)*	(11.46; 14.71)	(2.17; 4.14)	(49.62; 53.10)	(71.82; 77.38)
Ex-smoker	35.28	13.23	2.42	48.51	72.72
	(34.28; 36.27)	(12.34; 14.19)	(1.97; 2.87)	(47.33; 49.69)	(70.97; 74.39)
Smoker	31.50	11.82	1.73	43.32	72.72
	(30.47; 32.65)	(10.76; 12.95)	(1.29; 2.32)	(42.27; 44.56)	(70.54; 74.82)
Females					
Never smoker	36.99	19.21	5.51	56.20	65.82
	(36.06; 37.90)	(18.05; 20.65)	(4.78; 6.37)	(54.90; 57.71)	(63.95; 67.37)
Ex-smoker	34.09	19.52	4.53	53.60	63.59
	(32.75; 35.38)	(17.93; 21.45)	(3.55; 5.91)	(51.99; 55.73)	(61.05; 66.04)
Smoker	30.73	17.29	3.28	48.02	64.00
	(29.12; 32.59)	(15.36; 20.52)	(2.06; 5.60)	(46.31; 51.28)	(59.69; 67.43.)

*: 95% confidence interval.

**Table 5:** **Decomposition of the difference between ex- and current smokers with never smokers in Disability Free Life Expectancy (DFLE**_**30**_**), (Severe) Disability Life Expectancy (DLE(_S)**_**30**_**), Life Expectancy (LE**_**30**_**) at age 30 by type of effect (mortality or disability), Health Interview Survey 1997 and 2001 and follow-up until respectively 31/12/2007 and 31/12/2010, Belgium**

	**DFLE**_**30**_			**DLE**_**30**_			**DLE_S**_**30**_			**LE**_**30**_		
**Smoking status**	**Difference**	**Mortality effect**	**Disability effect**	**Difference**	**Mortality effect**	**Disability effect**	**Difference**	**Mortality effect**	**Disability effect**	**Difference**	**Mortality effect**	**Disability effect**
Males												
Ex-smoker	-3.02	-1.13	-1.89	0.34	-1.55	1.89	-0.59	-0.58	-0.01	-2.68	-2.68	0
	(-4.87; -1.34)^*^	(-2.13; -0.19)	(-3.29; -0.42)	(-1.61; 2.08)	(-2.89; -0.40)	(0.42; 3.29)	(-1.77; 0.36)	(-1.26; -0.11)	(-0.93; 0.80)	(-4.88; -0.78)	(-4.88; -0.78)	
Smoker	-6.80	-3.67	-3.13	-1.07	-4.21	3.13	-1.27	-1.38	0.11	-7.87	-7.87	0
	(-8.64; -4.96)	(-4.84; -2.63)	(-4.65; -1.53)	(-3.32; 0.87)	(-5.78; -2.96)	(1.53; 4.65)	(-2.49; -0.23)	(-2.30; -0.82)	(-0.92; 1.10)	(-10.27; -5.35)	(-10.27; -5.35)	
Females												
Ex-smoker	-2.90	-0.74	-2.16	0.31	-1.85	2.16	-0.98	-0.84	-0.14	-2.59	-2.59	0
	(-4.46; -1.29)	(-1.49; -0.04)	(-3.62; -0.12)	(-1.82; 2.62)	(-3.49; -0.12)	(0.63; 3.62)	(-2.24; 0.48)	(-1.66; 0.10)	(-1.31; 1.09)	(-4.69; -0.36)	(-4.69; -0.36)	
Smoker	-6.25	-2.49	-3.77	-1.92	-5.69	3.77	-2.23	-2.43	0.19	-8.17	-8.17	0
	(-8.17; -4.24)	(-3.67; -1.45)	(-5.78; -1.50)	(-4.30; 1.27)	(-7.41; -3.06)	(1.50; 5.78)	(-3.85; 0.26)	(-3.72; -1.00)	(-1.89; 2.55)	(-10.40; -4.86)	(-10.40; -4.86)	

*: 95% confidence interval.

**Figure 1:**
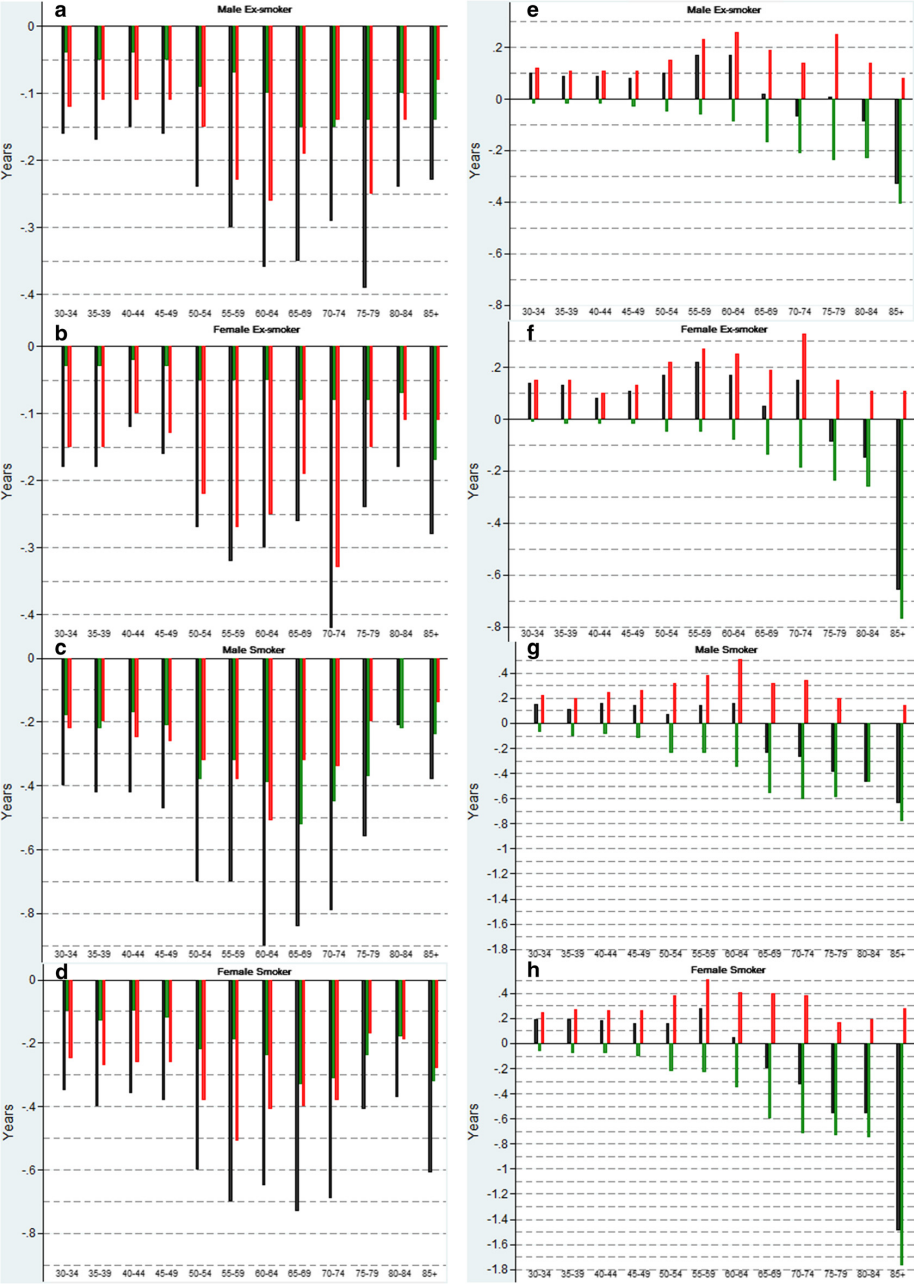
**Decomposition by age of the difference between ex- and current smokers with never smokers in Disability Free Life Expectancy (DFLE**_**30**_**), Disability Life Expectancy (DLE**_**30**_**) at age 30 and type of effect (mortality or disability), Health Interview Survey 1997 and 2001 and follow-up until respectively 31/12/2007 and 31/12/2010, Belgium**. Legend: Panels a-d: DFLE (**a**: Male Ex-smoker; **b**: Female Ex-smoker; **c**: Male Smoker; **d**: Female Smoker). Panels **e-h**: DLE (**e**: Male Ex-smoker; **f**: Female Ex-smoker; **g**: Male Smoker; **h**: Female Smoker). Black bar: difference DFLE or DLE with never smokers. Green bar: mortality effect. Red bar: disability effect. E.g. black bar in panel **a**: DFLE among males Ex-smokers minus DFLE among males never smokers; black bar in panel **h**: DLE among females Ex-smokers minus DLE among females never smokers.

**Figure 2:**
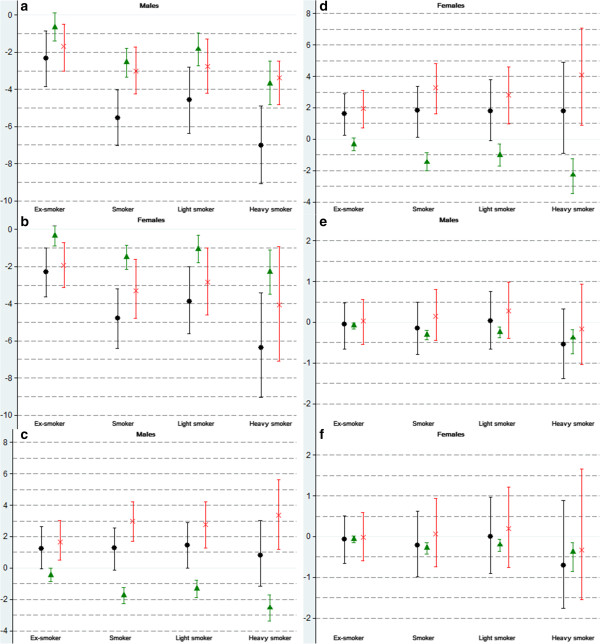
**Decomposition of the difference between ex- and current smokers with never smokers in Disability Free Life Expectancy (DFLE**_**30–80**_**), (Severe) Disability Life Expectancy (DLE(_S)**_**30–80**_**) between ages 30 and 80 by type of effect (mortality or disability), Health Interview Survey 1997 and 2001 and follow-up until respectively 31/12/2007 and 31/12/2010, Belgium.** Legend: Black dot symbol: difference DFLE, DLE or DLE_S with never smoker and 95% CI. Green triangle symbol: mortality effect and 95% CI. Red letter x symbol: disability effect and 95% CI.

Figure 1 presents the decomposition by age of (1) the difference in DFLE and DLE between never smokers and (ex-)smokers and of (2) the mortality and disability component of these differences. The disability effect is the most important contributor to the shorter DFLE among ex-smokers up to the age of 84 years (Figure 1a-b) and up to age 64 years and 74 years for male and female smokers respectively (Figure 1c-d). For the difference in DLE between never smokers and (ex-)smokers, the disability effect is actually outweighed by the mortality effect only in the older ages: 70+ years and 75+ years for male and female ex-smokers respectively, and age 65+ years for male and female smokers. For ex-smokers, the largest proportion (67%) of the disability effect of DLE difference is concentrated before age 70 years while for male and female smokers the proportion of the disability effect before age 70 years is 78% and 73% respectively (Figure 1e-h). At young ages, the importance of the disability disadvantage to the longer DLE in ex-smokers, smokers, light smokers and heavy smokers is further shown by the decomposition of the difference in the partial DLE in the age window 30 to 80 years (DLE_30–80_) (Tables 6, Figure 2). Within this age window, any smoking category experiences more years with disability compared to never smokers, as the disability effect cancels out the mortality effect. For example, the difference in DLE_30–80_ among male ex-smokers compared to never smokers is 1.22 years (1.22 years (95% CI: -0.04; 2.62) = -0.45 years (mortality effect) + 1.67 (disability effect)). The difference with smokers is 1.27 years (95% CI: -0.13; 2.57). We observe a larger difference among light males smokers (1.45 years (95% CI: -0.02; 2.90)) compared to difference among heavy smokers (0.82 years (95% CI: -1.15; 3.01)) suggesting a larger contribution of the mortality effect for heavy smokers even before age 80 years old. The difference in DLE_30–80_ among female ex-smokers, smokers, light and heavy smokers compared to never smokers is respectively 1.62 years (95 CI; 0.26; 2.88), 1.83 years (95 CI; 0.13; 3.35), 1.80 years (95 CI; -.0.09; 3.78) and 1.80 years (95 CI; -0.90; 4.90). Restricting the analysis to severe disability, the mortality effect by far outweighs any disability effect and is the most important contributor to shorter DLE_S_30–80_ in any age group. None of the differences in DLE_S_30–80_ for the different smoking categories compared to never smokers is statistically significant (Figure 2).

**Table 6:** **Disability Free Life Expectancy (DFLE**_**30–80**_**), (Severe) Disability Life Expectancy (DLE(_S)**_**30–80**_**), Life Expectancy (LE**_**30–80**_**) and the % of remaining life without disability (% DFLE/LE**_**30–80**_**) between ages 30 and 80 by smoking status, Health Interview Survey 1997 and 2001 and follow-up until respectively 31/12/2007 and 31/12/2010, Belgium**

**Smoking status**	**DFLE**_**30–80**_	**DLE**_**30–80**_	**DLE_S**_**30–80**_	**LE**_**30–80**_	**% DFLE/LE**_**30–80**_
Males					
Never smoker	36.38	9.46	1.52	45.84	79.37
	(35.22; 37.54)*	(8.37; 10.59)	(1.06; 2.04)	(44.97; 46.62)	(76.93; 81.65)
Ex-smoker	34.06	10.68	1.47	44.74	76.13
	(33.09; 35.01)	(9.90; 11.48)	(1.17; 1.82)	(43.84; 45.56)	(74.39; 77.79)
Smokers	30.85	10.72	1.38	41.58	74.21
	(29.87; 31.88)	(9.76; 11.58)	(0.97; 1.80)	(40.66; 42.19)	(72.24; 76.36)
Light smoker	31.80	10.90	1.56	42.70	74.46
	(30.53; 33.05)	(9.82; 12.01)	(1.09; 2.09)	(41.55; 43.82)	(71.96; 76.86)
Heavy smoker	29.35	10.28	0.98	39.63	74.06
	(27.53; 31.23)	(8.70; 11.94)	(0.39; 1.78)	(38.16; 41.10)	(70.09; 78.01)
Females					
Never smoker	34.77	12.47	2.22	47.23	73.60
	(33.97; 35.49)	(11.82; 13.25)	(1.87; 2.58)	(46.70; 47.75)	(72.02; 74.91)
Ex-smoker	32.48	14.08	2.15	46.57	69.76
	(31.35; 33.56)	(12.98; 15.23)	(1.67; 2.65)	(45.76; 47.32)	(67.38; 71.97)
Smokers	29.98	14.30	2.02	44.28	67.70
	(28.66; 31.50)	(12.79; 15.60)	(1.35; 2.75)	(43.27; 45.33)	(64.85; 71.02)
Light smoker	30.90	14.27	2.22	45.17	68.40
	(29.19; 32.61)	(12.53; 16.01)	(1.43; 3.10)	(43.91; 46.46)	(64.83; 72.15)
Heavy smoker	28.41	14.27	1.52	42.68	66.56
	(25.77; 31.25)	(11.54; 17.29)	(0.56; 3.09)	(40.73; 44.65)	(60.22; 72.71)

*: 95% confidence interval.

## Discussion

The study confirms that smoking kills but also shows that smoking increases the years lived with disability before age 80 years old, while at older ages, the excess mortality of smokers hides the smoker disability disadvantage. In other words, through the excess premature mortality of smokers, their DLE is shorter compared to never smokers. When the intensity of smoking is high, the excess mortality hides the disability disadvantage in DLE even before age 80. Our study also shows that ex-smokers have a shorter DFLE and a longer DLE. The disability disadvantage that ex-smokers have is the main contributor to the shorter DFLE and longer DLE compared to never smokers, even though mortality rates for ex-smokers may approach those for never smokers. At older ages, as for smokers, the excess mortality offsets the disability disadvantage but this occurs at an older age than for smokers.

So at the one hand, the observations support the expansion hypothesis: in the end smokers may live less years with disability due to their strong excess mortality. At the other hand, ex-smokers and smokers have to endure more years with disability before age 80 years. These seemingly two opposing observations are a result of the fact that the expression of the difference in disability prevalence are concentrated at the younger ages, while the strength of smoking related mortality disadvantage is greater at older ages and reduces the years to be lived with and without disability. Moreover, the interaction between excess mortality and excess disability is further a function of gender, smoking intensity and the severity level of the disability. The expression of the disability effect is somewhat higher in women for whom lower premature excess mortality reduces DLE less than for men. The female–male difference in the mortality and disability impact of smoking may be a contributing factor to the gender health-survival paradox [35]. Our study also suggests a significantly shorter LE free of severe disability (DFLE_S) for male heavy smokers compared to never smokers.

Overall, our study does support the statement that smoking is associated with mortality more than with disability, and that through excess mortality the years of life with disability are compressed compared to never smokers [15-17]. However the findings also partly corroborate previous reports [18-20] suggesting that smoking has an important and distinct impact on disability which results in more years with disability at younger ages for ex-smokers and smokers. Other studies on the effect of smoking have also reported an increased incidence of disability, a lower (physical) health related quality of life and an elevated use of health care services [11,12,36-39].

Our analysis has several strengths. We were able to use one data set which had smoking, disability and mortality data. For the mortality follow-up of the survey, less than 3% of the participants could not be linked to the National Register. Our decomposition analysis allowed division of the differences in DFLE and DLE into the part due to excess mortality for (ex-)smokers and the part due to their excess disability, as well as how these varied by age group. This therefore helped explain the controversy that longer LE for non-smokers compared to (ex-)smokers translates into more years of disability. To obtain further insight we evaluated in which age groups the mortality effect or the disability effect were more substantial. To our knowledge, this paper is the first to show the excess disability associated with smoking contributing to more years with disability at younger ages.

Limitations of the study are related to the cross-sectional design providing the smoking and disability data. E.g. current smokers at any age after the age of 30 years may be considered as lifelong smokers as the likelihood of smoking initiation after the age of 30 years is small. If we ignore non-successful smoke stop attempts, the category “current smoker” is probably a less heterogeneous population compare to the category of ex-smokers for whom no information on the age or the time since they stop smoking and their reasons to stop smoking is used: health benefits are larger in early quitters while former smokers who recently quit tend to have more health problems [6,36]. Further, we cannot attribute the lower prevalence of disability (which led among never smokers to more healthy years and to a reduction in the time spent with disability before age 80) to either a lower disability incidence or a higher recovery rate since this is beyond the decomposition method using Sullivan method based estimates [33]. The main assumption of stationary population in order to minimise bias of the Sullivan method compared to the multistate life table method using transition probability may hold as changes in smoking behaviour do not lead to sudden changes in both mortality incidence and disability incidence [40]. It is difficult to identify to what extend the method used to estimate the years lived with and without disability contributes to the lack of agreement related to the compression of disability in function of smoking elimination. Some authors include both transitions to disability and recovery [14,16,20] in the multistate method, others do not [17]. Next, studies differ further in definition of disability, the definition of smoking categories. Studies also studies differ in the age the DFLE and LE is estimated. The paper of Nusselder et al. [20], is the only one using the multistate method, including both disability incidence and recovery transitions, that provides evidence for a compression of years with disability related to smoking elimination both at age 30 and at age 70 years. The same conclusion was made by Bronnum-Hansen et al. using the Sullivan method [19]. Other studies using a multistate approach report that smoking reduces both the duration of life with and without disability [14,16,17].

Secondly, low survey participation may bias the results [41]. We have shown in prior studies that participation is differentially linked to health status and socioeconomic position [42,43]. Charafedinne R. et al. [44] compared Belgian census-based DFLE by social position with survey-based estimates and found that although there was no statistical difference, the difference in LE and DFLE should be acknowledged. Low educated survey participants tended to be less healthy (i.e. having a lower LE and lower DFLE) compared to their counterparts in the general population, while the inverse was observed in the highest educational groups. The same author also reported evidence supporting the hypothesis that educational attainment does not substantially influence the association between smoking and mortality [28]. Therefore, we hypothesize that any selection bias in the difference in DFLE or DLE by smoking is most likely related to the survey-based disability prevalence and not to the mortality. If any, it is expected to overestimate the gap and the disability effect of the smoking related differences in DFLE and DLE.

Other limitations are related to the validity of survey data. The validity of self-reported smoking can be questioned, although a number of studies have found the validity of this self-reporting high [45]. However we expect that any misclassification of smoking status would result in underestimation of the reported differences. A final important limitation is related to the delay in coding causes of mortality in Belgium. We were not able to estimate the contribution of specific diseases to the differences in DFLE and DLE by smoking status. This limits the interpretation on the role of specific diseases interfering with the balance between the smoking related excess of mortality and the smoking related disability.

## Conclusion

We were able to evaluate the contribution of the excess mortality versus the disabling impact of tobacco exposure on population health. Smoking kills and shortens both life without and with disability mainly due to its related excess mortality. However excess disability associated with smoking cannot be ignored given its contribution to substantially more years with disability before age 80.

The important population health message remains: smoking is a major health hazard. Policy on smoking should strive for a smoke-free society through primordial prevention or reduction of smoking initiation and through primary prevention or smoke stop to increase LE and DFLE. Further, given the lack of compression of disability for never smokers compared to smokers, this study highlight the need for policy makers to monitor not only DFLE (e.g. the European Union 2020 health goal to increase the healthy and active ageing of the European population by 2 years [46]) but also DLE as reduction in health risks and the increase in DFLE, may not automatically result in a simultaneous reduction or status quo of the DLE.

## Abbreviations

ADL: Activities of daily living; a-PR: Adjusted prevalence ratio; CI: Confidence interval; DFLE: Disability free life expectancy; DLE: Disability life expectancy; DLE_S: Severe disability life expectancy; HIS: Health interview survey; ISCED: International Standard Classification of Education; LE: Life expectancy.

## Competing interests

None of the authors have to declare financial or non-financial competing interest.

## Authors’ contributions

HVO worked out the concept and design of the study, performed part of the statistical analysis, participated in the interpretation; and drafted the manuscript. NB performed part of the statistical analysis, participated in the interpretation; and in the drafting of the manuscript. WN, CJ, EC and JMR participated in the concept development and the design of the study, the interpretation and revision of the manuscript. RC participated in the statistical analysis, the interpretation and revision of the manuscript. SD participated in the interpretation and the revision of the manuscript. All authors read and approved the final manuscript.

## Pre-publication history

The pre-publication history for this paper can be accessed here:

http://www.biomedcentral.com/1471-2458/14/723/prepub

## References

[B1] World Health Organisation WHO Global Report: Mortality Attributable To Tobacco 2012 Geneva: WHO 1 396

[B2] Mathers CD Loncar D Projections of global mortality and burden of disease from 2002 to 2030 PLoS Med 2006 3 e442 17132052 10.1371/journal.pmed.0030442 PMC1664601

[B3] Jha P Ramasundarahettige C Landsman V Rostron B Thun M Anderson RN McAfee T Peto R 21st-century hazards of smoking and benefits of cessation in the United States N Engl J Med 2013 368 341 350 23343063 10.1056/NEJMsa1211128

[B4] Sakata R McGale P Grant EJ Ozasa K Peto R Darby SC Impact of smoking on mortality and life expectancy in Japanese smokers: a prospective cohort study BMJ 2012 345 e7093 23100333 10.1136/bmj.e7093 PMC3481021

[B5] Pirie K Peto R REEVES GK Green J Beral V The 21st century hazards of smoking and benefits of stopping: a prospective study of one million women in the UK Lancet 2013 381 133 141 23107252 10.1016/S0140-6736(12)61720-6 PMC3547248

[B6] Frosch ZAK Dierker LC Rose JS Waldinger RJ Smoking trajectories, health, and mortality across the adult lifespan Addict Behav 2009 34 701 704 19428188 10.1016/j.addbeh.2009.04.007 PMC2700828

[B7] Levy DT Ellis JA Mays D Huang AT Smoking-related deaths averted due to three years of policy progress Bull World Health Organ 2013 91 509 518 23825878 10.2471/BLT.12.113878 PMC3699793

[B8] Al Mamun A Peeters A Barendregt J Willekens F Nusselder W Bonneux L Smoking decreases the duration of life lived with and without cardiovascular disease: a life course analysis of the Framingham Heart Study Eur Heart J 2004 25 409 415 15033253 10.1016/j.ehj.2003.12.015

[B9] Devereux G ABC of chronic obstructive pulmonary disease. Definition, epidemiology, and risk factors BMJ 2006 332 1142 1144 16690673 10.1136/bmj.332.7550.1142 PMC1459603

[B10] Lee PN Forey BA Coombs KJ Systematic review with meta-analysis of the epidemiological evidence in the 1900s relating smoking to lung cancer BMC Cancer 2012 12 385 22943444 10.1186/1471-2407-12-385 PMC3505152

[B11] Artaud F Dugravot A Sabia S Singh-Manoux A Tzourio C Elbaz A Unhealthy behaviours and disability in older adults: Three-City Dijon cohort study BMJ 2013 347 f4240 23881930 10.1136/bmj.f4240

[B12] Strandberg AY Strandberg TE Pitkälä K Salomaa VV Tilvis RS Miettinen TA The effect of smoking in midlife on health-related quality of life in old age: a 26-year prospective study Arch Intern Med 2008 168 1968 1974 18852397 10.1001/archinte.168.18.1968

[B13] Belanger A Martel L Berthelot JM Wilkins R Gender differences in disability-free life expectancy for selected risk factors and chronic conditions in Canada J Women Aging 2002 14 61 83 12537280 10.1300/J074v14n01_05

[B14] Ferrucci L Izmirlian G LEVEILLE S Phillips CL Corti MC Brock DB Guralnik JM Smoking, physical activity, and active life expectancy Am J Epidemiol 1999 149 645 653 10192312 10.1093/oxfordjournals.aje.a009865

[B15] Klijs B Mackenbach JP Kunst AE Obesity, smoking, alcohol consumption and years lived with disability: a Sullivan life table approach BMC Public Health 2011 11 378 21605473 10.1186/1471-2458-11-378 PMC3128016

[B16] Majer IM Nusselder WJ Mackenbach JP Kunst AE Life expectancy and life expectancy with disability of normal weight, overweight, and obese smokers and nonsmokers in Europe Obes 2011 19 1451 1459 10.1038/oby.2011.46 21415846

[B17] Reuser M Bonneux L Willekens F Smoking kills, obesity disables: a multistate approach of the U.S. Health and Retirement Survey Obesity 2009 17 783 789 19165165 10.1038/oby.2008.640

[B18] Bronnum-Hansen H Juel K Abstention from smoking extends life and compresses morbidity: a population based study of health expectancy among smokers and never smokers in Denmark Tob Control 2001 10 273 278 11544393 10.1136/tc.10.3.273 PMC1747595

[B19] Bronnum-Hansen H Juel K Davidsen M Sorensen J Impact of selected risk factors on expected lifetime without long-standing, limiting illness in Denmark Prev Med 2007 45 49 53 17467783 10.1016/j.ypmed.2007.03.010

[B20] Nusselder WJ Looman CWN Marang-van de Mheen PJ van de Mheen H Mackenbach JP Smoking and the compression of morbidity J Epidemiol Community Health 2000 54 566 574 10890867 10.1136/jech.54.8.566 PMC1731729

[B21] Gruenberg E The failure of success The Milbank Memorial Fund Quarterly/Health Soc 1977 55 3 24 141009

[B22] Fries JF Bruce B Chakravarty E Compression of morbidity 1980–2011: a focused review of paradigms and progress J Aging Res 2011 2011 261702 21876805 10.4061/2011/261702 PMC3163136

[B23] Demarest S Van der Heyden J Charafeddine R Drieskens S Gisle L Tafforeau J Methodological basics and evolution of the Belgian Health Interview Survey 1997–2008 Arch Public Health 2013 71 24 24047278 10.1186/0778-7367-71-24 PMC3844891

[B24] de Bruin A Picavet HS Nossikov A Health Interview Surveys: Towards International Harmonization of Methods and Instruments 1996 World Health Organisation: Copenhagen 8857196

[B25] Ware JE Sherbourne CD The MOS 36-item short-form health survey (SF-36). I. Conceptual framework and item selection Med Care 1992 30 473 483 1593914

[B26] Unesco Institute for Statistics International Standard Classification of Education, ISCED 2011 2012 Montreal: UNESCO 1 88

[B27] Kirkwood B Sterne J Kirkwood B, Sterne J Poisson Regression Essential Medical Statistics 2003 second Malden, MA: Blackwell Science 249 262

[B28] Charafeddine R Van Oyen H Demarest S Does the association between smoking and mortality differ by socioeconomic status? Soc Sci Med 2012 74 1402 1406 22401648 10.1016/j.socscimed.2012.01.015

[B29] Charafeddine R Demarest S Van der Heyden J Tafforeau J Van Oyen H Using multiple measures of social inequalities to study time trends in smoking inequalities Eur J Publ Health 2012 23 546 551 10.1093/eurpub/cks083 22711785

[B30] Jagger C Cox B Le Roy S Clavel A Robine JM Romieu I Van Oyen H Health Expectancy Calculation by the Sullivan Method: A Practical Guide 2007 Third (http://www.eurohex.eu/pdf/Sullivan_guide_final_jun2007.pdf). EHEMU Technical report 2006_3, 1-44. 2007. Montpellier, France

[B31] Sullivan DF A single index of mortality and morbidity HSMHA Health Rep 1971 86 347 354 5554262 PMC1937122

[B32] Nusselder W Jagger C Cox B Cambois E Van Oyen H Robine JM Doblhammer G Rychtarikova J Gillies C Weston C Kruse A Beluche I Counienc R Hassen-Khodja C Romieu I Perrier C WP7: Decomposition Tools. Technical Report on Decomposition (http://www.eurohex.eu/pdf/Reports_2010/2010TR7.1_Decomposition%20tools.pdf). EHEMU Technical report 2010_7.1, 1–49. 2010. Montpellier, France

[B33] Nusselder WJ Looman CW Decomposition of differences in health expectancy by cause Demography 2004 41 315 334 15209043 10.1353/dem.2004.0017

[B34] Robine JM Cambois E Nusselder W Jeune B Van Oyen H Jagger C The joint action on healthy life years (JA: EHLEIS) Arch Public Health 2013 71 2 23379576 10.1186/0778-7367-71-2 PMC3598905

[B35] Van Oyen H Nusselder W Jagger C Kolip P Cambois E Robine JM Gender differences in healthy life years within the EU: an exploration of the “health-survival” paradox Int J Public Health 2013 58 143 155 22618297 10.1007/s00038-012-0361-1 PMC3557379

[B36] Azagba S Sharaf MF Xiao LC Disparities in health care utilization by smoking status in Canada Int J Public Health 2013 58 913 925 23436022 10.1007/s00038-013-0452-7

[B37] Holahan CK Holahan CJ North RJ Hayes RB Powers DA Ockene JK Smoking status, physical health-related quality of life, and mortality in middle-aged and older women Nicotine Tob Res 2013 15 662 669 22965789 10.1093/ntr/nts182 PMC3611990

[B38] Laaksonen M Rahkonen O Martikainen P Karvonen S Lahelma E Smoking and SF-36 health functioning Prev Med 2006 42 206 209 16443264 10.1016/j.ypmed.2005.12.003

[B39] Nolte E Mckee M Variations in amenable mortality–trends in 16 high-income nations Health Policy 2011 103 47 52 21917350 10.1016/j.healthpol.2011.08.002

[B40] Mathers CD Robine JM How good is Sullivan’s method for monitoring changes in population health expectancies J Epidemiol Community Health 1997 51 80 86 9135793 10.1136/jech.51.1.80 PMC1060414

[B41] Galea S Tracy M Participation rates in epidemiologic studies Ann Epidemiol 2007 17 643 653 17553702 10.1016/j.annepidem.2007.03.013

[B42] Demarest S Van der Heyden J Charafeddine R Tafforeau J Van Oyen H Van Hal G Socio-economic differences in participation of households in a Belgian national health survey Eur J Publ Health 2012 23 981 985 10.1093/eurpub/cks158 23183496

[B43] Lorant V Demarest S Miermans PJ Van Oyen H Survey error in measuring socio-economic risk factors of health status: a comparison of a survey and a census Int J Epidemiol 2007 36 1292 1299 17898025 10.1093/ije/dym191

[B44] Charafeddine R Berger N Demarest S Van Oyen H Using mortality follow-up of surveys to estimate social inequalities in healthy life years Popul Health Metrics 2014 12 13 10.1186/1478-7954-12-13 PMC4030465 24855457

[B45] Vartiainen E Seppala T Lillsunde P Puska P Validation of self reported smoking by serum cotinine measurement in a community-based study J Epidemiol Community Health 2002 56 167 170 11854334 10.1136/jech.56.3.167 PMC1732104

[B46] Lagiewka K European innovation partnership on active and healthy ageing: what have been the policy drivers and determinants to set a headline target of 2 additional Healthy Life Years at birth at EU average by 2020? Arch Public Health 2012 70 23 23088612 10.1186/0778-7367-70-23 PMC3492155

